# MSABrowser: dynamic and fast visualization of sequence alignments, variations and annotations

**DOI:** 10.1093/bioadv/vbab009

**Published:** 2021-08-07

**Authors:** Furkan M Torun, Halil I Bilgin, Oktay I Kaplan

**Affiliations:** 1 Rare Disease Laboratory, School of Life and Natural Sciences, Abdullah Gul University, Kayseri 38080, Turkey; 2 Department of Computer Engineering, Abdullah Gul University, Kayseri 38080, Turkey

## Abstract

**Summary:**

Sequence alignment is an excellent way to visualize the similarities and differences between DNA, RNA or protein sequences, yet it is currently difficult to jointly view sequence alignment data with genetic variations, modifications such as post-translational modifications and annotations (i.e. protein domains). Here, we present the MSABrowser tool that makes it easy to co-visualize genetic variations, modifications and annotations on the respective positions of amino acids or nucleotides in pairwise or multiple sequence alignments. MSABrowser is developed entirely in JavaScript and works on any modern web browser at any platform, including Linux, Mac OS X and Windows systems without any installation. MSABrowser is also freely available for the benefit of the scientific community.

**Availability and implementation:**

MSABrowser is released as open-source and web-based software under MIT License. The visualizer, documentation, all source codes and examples are available at https://thekaplanlab.github.io/ and GitHub repository https://github.com/thekaplanlab/msabrowser.

**Supplementary information:**

Supplementary data are available at *Bioinformatics Advances* online.

## 1 Introduction

The next-generation sequencing technologies have revolutionized the genomics field, thus revealing more than 700 million genetic variations in the human genomes and millions of genetic variants in non-human primates (Karczewski *et al.*, 2020; Locke *et al.*, 2011; Rhesus Macaque Genome Sequencing and Analysis Consortium *et al.*, 2007; Sherry *et al.*, 1999; Sundaram *et al.*, 2018; Taliun *et al.*, 2019; The Marmoset Genome Sequencing and Analysis Consortium, 2014). Furthermore, clinical scientists and researchers have identified thousands of variants associated with health and diseases. Additionally, genome-wide association studies systematically identified candidate genomic regions responsible for phenotypic differences (Landrum *et al.*, 2020; Ozaki *et al.*, 2002). All these data suggest that each genomic or proteomic position has a variety of unique details, including mutation, single-nucleotide polymorphism, allele frequency, disease associations, DNA methylation and amino acid phosphorylation at specific positions. Furthermore, non-human species, such as cat, dog, mice, cow, macaque, orangutan, pig, worm, opossum and zebrafish, have millions of variant records that are stored in organism-specific databases like Wormbase for *C*aenorhabditis *elegans*, Alliance of Genome Resources and Ensembl (Hunt *et al*., 2018; The Alliance of Genome Resources Consortium, 2020). Our recent work revealed that there are many identical variants called orthologous variants (OrthoVars) between humans and different species (Pir *et al.*, 2021). For example, RYR2 has a cysteine to tyrosine substitution at amino acid position 4957 in humans (Protein ID = NP 001026) and 4956 in mice (Protein ID = NP 076357) (Pir *et al.*, 2021), suggesting that p. C4957Y in human RYR2 is an OrthoVar of p. C4956Y in mice Ryr2. Furthermore, similar to human variants, many variants from non-human species have variant-specific annotations. For example, mice Tuba1a (Protein ID = NP_035783.1) contains a leucine to phenylalanine substitution at amino acid position 5, and mice with the p. I5F variation were produced via ENU mutagenesis and are viable as a heterozygote, suggesting that this variant has two specific annotations (Pham *et al*., 2019). Interestingly, mice Tuba1a (p.I5F) has an OrthoVar (p.I5L) in human TUBA1A, and the human TUBA1A(p.I5L) variant is implicated in a condition called polymicrogyria (Pham *et al*., 2019; Pir *et al.*, 2021). Finally, because CRISPR has been widely used to create OrthoVars in model organisms like mice, zebrafish, Drosophila and *C. elegans*, and because OrthoVars have grown in popularity as a result of their utility in understanding the functional interpretation of human genetic variants, co-visualizing OrthoVars from diverse organisms with variant-specific annotations (the clinical significance of variant, phenotypic data, etc.) would facilitate comparison of human variants and variant-specific annotations with variants and variant-specific annotations from non-human species (Arno *et al.*, 2016; Farr *et al*., 2018; Harnish *et al.*, 2019; Lin *et al*., 2016; Wong *et al.*, 2019). However, existing visualization tools for pair sequence alignment (PSA) and multiple sequence alignment (MSA) cannot easily incorporate position-specific annotations into the corresponding sequence positions on PSA and MSA.

**Fig. 1.**
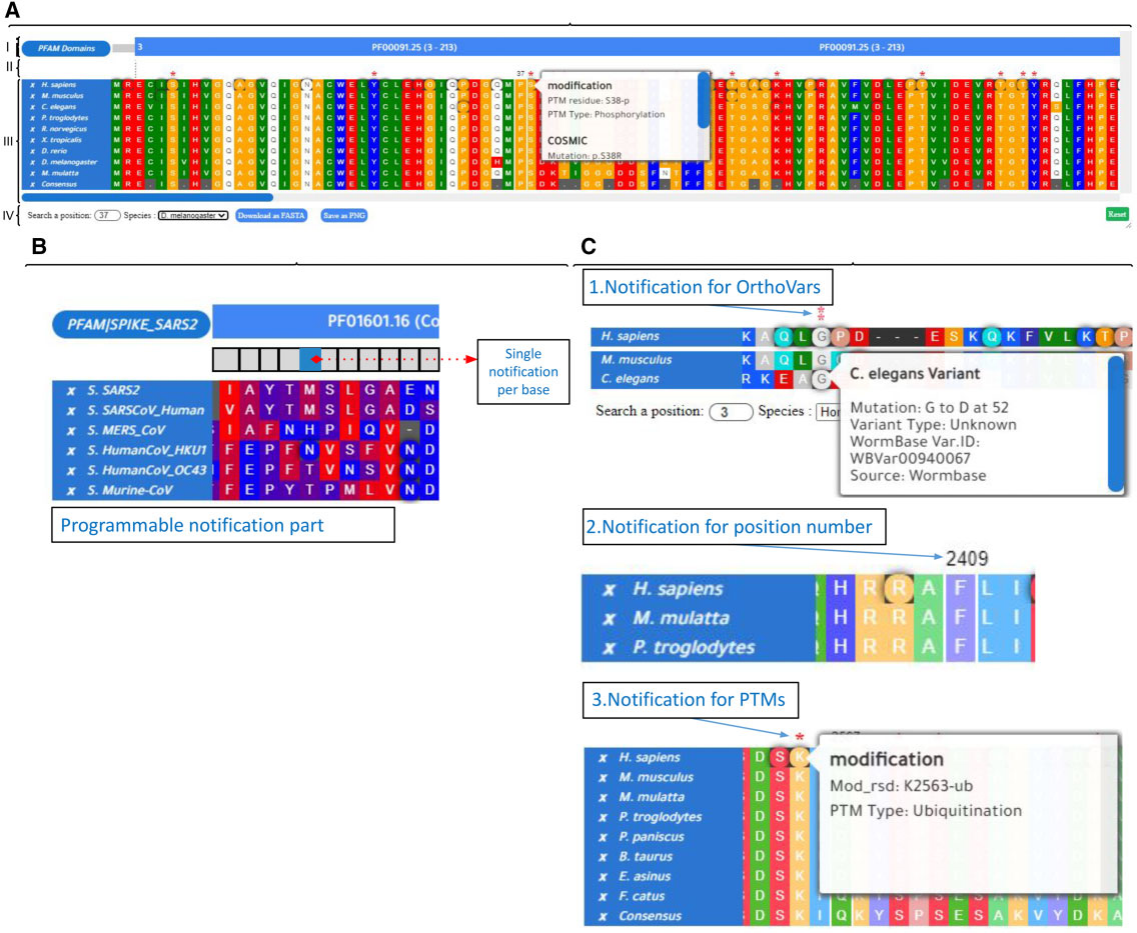
An overview of the MSABrowser tool. MSA for homologous proteins of the human TUBA1A is depicted in this figure, along with genetic variations on the corresponding positions on sequences and associated intervals such as protein domains. **(A) (I)** The annotation part represents the specified intervals for the sequence and in this example, it is used for illustrating the positions of the protein domains with cross-link features that enable users to locate the website or page of the original database or article. **(II)** The notification part shows any type of defined modifications as a red asterisk above the sequence per position and displays the searched position in a species above the alignments. **(III)** The sequence alignment part contains the imported alignment data with the previously selected colour scheme. Also, rounded (circle) positions indicate that at least one genetic variation or modification exists in this position. A rectangular white background pop-up box appears when the mouse hovers the specific position in the sequence and the genetic variations and modifications are listed in this pop-up box. On the bottom, an auto-generated ‘Consensus’ sequence is displayed. On the left side, species names contain cross-reference links for referring to the dedicated page of the sequence according to its protein identifier such as a UniProt number and the near-white ‘x’ button enables users to hide the sequence from the alignment together with its identifier. **(IV)** A position in the sequence of any species listed in the alignment can be searched and the sequence alignment data in FASTA format can be downloaded with the blue button and visualization of alignment data can be exported as PNG format. Also, with the green ‘Reset’ button, it is available to reload the viewer. **(B)** Visualization of MSAs of six virus spike proteins with the MSABrowser tool. The positions with the annotations are marked in a circle, while the positions without annotations are displayed in a square. The full MSA comparisons with annotations can be found at our dedicated GitHub site https://thekaplanlab.github.io/ (**C)** Shown is the display of orthologous variants (OrthoVars), the positions of amino acid position or nucleotide, or PTMs with the programmable notification part of MSABrowser

Here we, therefore, develop a free, open-source, user-friendly web-based tool called MSABrowser to dynamically and rapidly visualize MSAs, with the integration of variant-specific annotations [the clinical significance, phenotypic relevance, OrthoVars, post-translational modifications (PTMs), variant ID, etc.] to the corresponding positions (e.g. p. C4957Y in human RYR2 and p. C4956Y in mice Ryr2) (Fig. 1). MSABrowser is based on a JavaScript programming language that enables users to construct interactive pages with complex features, so it works easily without installation on any modern web browser.

MSABrowser introduces four major novelties: first, the flexible annotation of genetic variants (c.88C>G or p.P30A), OrthoVars, nonsense variants (a stop codon) or PTMs (ubiquitination at Lysine 2563; K2563-ub) into the respective sequence positions on the PSA and MSA (Fig. 1A–C) (Pir *et al.*, 2021). For example, p. H565Y in human FARSB (Protein ID = human NP_005678.3) is an OrthoVar of p. H567Y in *C. elegans* FARS-3 (the orthologue of human FARSB; Protein ID = *C. elegans* NP_495785). These OrthoVars were inserted into the PSA of human FARSB and *C. elegans* FARS-3 at their respective sequence locations (Pir *et al.*, 2021). Second, multiple annotations, such as small insertions/deletions, protein domains (e.g. SH3 domains) and/or user-specified intervals, and CRISPR single guide RNA (sgRNA) targeting a particular region of a genome can be added at the same time to the corresponding positions; third, the variant-specific annotations, including phenotypic data, variant ID and allele frequency, can be integrated into the corresponding positions. For example, p. R79Q in ARL13B (Protein ID = NP_001167621.1) has several variant-specific annotations, including variant ID (rs121912606), an allele frequency (3.98e-6), predicted as a pathogenic variant, and disease association (causing Joubert syndrome) (Cantagrel *et al.*, 2008; Karczewski *et al.*, 2020), and all of these annotations can easily be co-viewed at the respective site. Finally, while MSABrowser can dynamically and quickly visualize sequence alignments, variations and annotations, scrolling through PSAs/MSAs, searching and custom styling are implemented, thus allowing for a quick search of a specific position in species (such as 4th position in the first protein or 68th position in the second genomic sequence). Because scrolling to specific positions is not always possible, MSABrowser provides a feature that directly navigates users to specific positions. Furthermore, MSABrowser allows users to expand the context to include an image, link or other components (HTML tag) in the pop-up box (Fig. 1A).

**Fig. 2.**
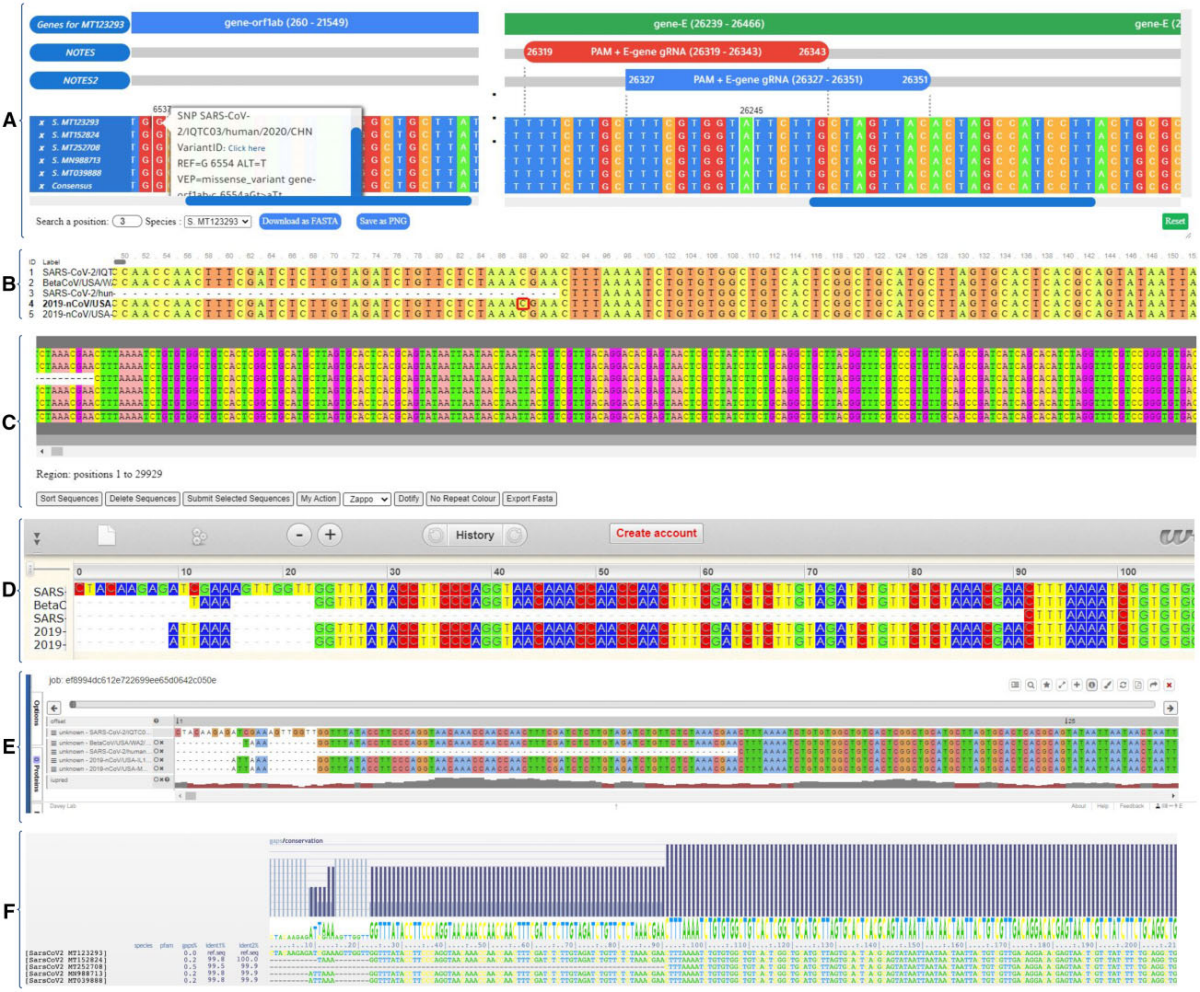
Comparison of MSA visualizers. The MSA of different genomes of the severe acute respiratory syndrome coronavirus 2 isolates (SARS-CoV-2: MT123293, MT152824, MT252708, MN988713 and MT039888) was created, followed by visualization with separate MSA viewers. **(A)** Shown is the MSA visualization with MSABrowser, which enables the addition of annotations (e.g. domains and notes) on top of the MSA. MSABrowser allows users to incorporate variant-specific annotations (missense variation, disease associations, variant ID, allele frequency, etc.). A pop-up will show up when users click on the circled amino acid or nucleotide position to display the annotations. Shown is a missense variation (G6537T) in SARS-CoV, an example of a particular annotation of a nucleotide position, MSABrowser enables users to remove the desired sequence by clicking the X button which appears in the far left of each line. Users can look up positions, download the FASTA and save the MSA as PNG. **(B)** Shown is MSAViewer tool on the same alignment as in A. Users can scroll to the left and right to see the rest of MSA. When the user clicks on a position, the amino acid is highlighted with a red square as in the position 88. **(C)** Shown is JSAV. It is possible to sort and delete sequences, add new sequences, change the colour schema and export FASTA with the buttons listed below the MSA. **(D)** Shown is Wasabi in which zoom in and zoom out options are enabled and scrolling is necessary to see the rest of the sequence. **(E)** Shows Proviz where users are able to search for a motif, switch to full screen, export the MSA and share it as a URL using the buttons located in the top right corner. **(F)** Shown is AlignmentViewer. For each sequence in the alignment, gaps ratio and identification ratio to the reference sequence is provided. Gaps and conservation per position are also shown above the MSA

While the MSABrowser can easily integrate annotations (OrthoVars, PTMs, allele frequency, variants, variant ID, etc.) into the corresponding positions, it is difficult or impossible for other MSA visualization tools (Fig. 2A–F and Supplementary Table S1). While some other tools also provide an option to highlight sequence intervals, it is not always possible to add multiple annotations. However, with MSABrowser, users can easily place all types of sequence intervals or highlight the regions (such as deletions, protein domains, CRISPR sgRNA, etc.) (Fig. 2A). Additionally, MSABrowser can also function as a stand-alone component separate from the rest of a website or database, and it can be easily embedded into a web page (Supplementary Table S1).

## 2 Availability and implementation

PSAs and MSAs are the fundamental methods for the alignment of any sequences of DNA, RNA and protein (Chenna, 2003; Higgins and Sharp, 1988). The MSABrowser imports PSA and MSA data in FASTA format with a file, and variations and sequence annotation data in JavaScript Object Notation (JSON) (Pearson, 1999). After parsing the alignment data and creating the consensus sequence, it then creates two main components: the annotation part and the sequence alignment part. For performance purposes, instead of rendering all the alignment data at once, the MSABrowser renders as the user navigates through the sequence alignment. The positions consisting of the modifications such as PTMs or variations are highlighted with shadow or asterisk together with rounded boxes on the corresponding positions of nucleotide or amino acids and hovering on them triggers a pop-up that shows the details of variations and modifications or any other provided notes for the position.

The MSABrowser has multiple ways of navigating the alignment. Firstly, by scrolling through the sequence alignment and secondly, by specifying either amino acid or nucleotide position and the species in the bottom panel. Users can hide sequences from the alignment by selection. Additionally, a cross-reference link is automatically generated based on the sequence identifiers from the imported FASTA file. Therefore, users may click the species names to jump to the sequence database (i.e. Ensembl, NCBI and UniProt). For visualizing the alignments, users might choose between 13 predefined colour schemes. The MSABrowser is capable of exporting alignment as a FASTA file format and the visualization as a publication-quality figure in Portable Network Graphics (PNG). Furthermore, we provide a detailed comparison of features among other visualization tools (Hossain, 2019; Jehl *et al.*, 2016; Larsson, 2014; Martin, 2014; Veidenberg *et al.*, 2016; Waterhouse *et al*., 2009; Yachdav *et al.*, 2016) in Supplementary Table S1 (Fig. 2).

## 3 Conclusion

MSABrowser is the most recently created tool that allows the visualization of MSAs, genetic variations, PTMs and protein domains at the same time. MSABrowser makes it much easier to display orthologous variants between different species (Pir *et al.*, 2021). Importantly, it does not require the installation of any software as it runs on any modern browser that is pre-installed on computers. Due to its portability, speed and ease of use, MSABrowser will be useful as a visualization tool for sequence alignment, variations and annotations for the scientific community.

## Supplementary Material

vbab009_Supplementary_Data
